# Age-Dependence of Flow Homeostasis in the Left Ventricle

**DOI:** 10.3389/fphys.2019.00485

**Published:** 2019-04-26

**Authors:** Yolanda Benito, Pablo Martinez-Legazpi, Lorenzo Rossini, Candelas Pérez del Villar, Raquel Yotti, Yolanda Martín Peinador, Daniel Rodríguez-Pérez, M. Mar Desco, Constancio Medrano, Jose Carlos Antoranz, Francisco Fernández-Avilés, Juan C. del Álamo, Javier Bermejo

**Affiliations:** ^1^Department of Cardiology, Hospital General Universitario Gregorio Marañón, Facultad de Medicina, Universidad Complutense de Madrid, Instituto de Investigación Sanitaria Gregorio Marañón and CIBERCV, Madrid, Spain; ^2^Department of Mechanical and Aerospace Engineering, University of California, San Diego, La Jolla, CA, United States; ^3^Centro de Salud Goya, Dirección Asistencial Centro, Atención Primaria de Madrid, Madrid, Spain; ^4^Department of Mathematical Physics and Fluids, Facultad de Ciencias, Universidad Nacional de Educación a Distancia, Madrid, Spain; ^5^Institute for Engineering in Medicine, University of California, San Diego, La Jolla, CA, United States

**Keywords:** Doppler-echocardiography, intraventricular flow, intraventricular flow patterns, blood stasis, hemodynamic shear stress, thrombosis

## Abstract

**Background:** Intracardiac flow homeostasis requires avoiding blood stasis and platelet activation during its transit through the cardiac chambers. However, the foundations of intraventricular blood washout and its exposure to shear stresses have been poorly addressed. We aimed to characterize and quantify these features in a wide population of healthy subjects and assess the relationships of these indices with age.

**Methods:** We used color-Doppler echocardiography and custom post-processing methods to study 149 healthy volunteers from 26 days to 80 years old. From the intraventricular flow-velocity fields we obtained personalized maps of (1) the residence time of blood in the LV, and (2) the shear index, a metric accounting for the strongest occurrence of shear stresses inside the chamber. From these maps we derived quantitative indices of the overall intraventricular blood washout and shear exposure. We addressed the age-dependence of these indices and analyzed their relationship with age-related changes in filling-flow.

**Results:** The entire intraventricular blood pool was replaced before 8 cycles. Average residence time of blood inside the LV was <3 cycles in all subjects and followed an inverse U-shape relationship with age, increasing from median (IQR) of 1.0 (0.7 to 1.2) cycles in the 1st year of life to 1.8 (1.4–2.2) cycles in young adults (17–30 years old), becoming shorter again thereafter. Shear index showed no relation with age and was bounded around 20 dyn·s/cm^2^. Regions with the longest residence time and highest shear index were identified near the apex. Differences in the degree of apical penetration of the filling waves and the duration of the late-filling phase explained the age-dependence of residence time (${\text{R}}_{\text{adj}}^{2}$ = 0.48, *p* < 0.001).

**Conclusions:** In average, blood spends 1 to 3 beats inside the LV with very low shear stress rates. The apical region is the most prone to blood stasis, particularly in mid-aged adults. The washout of blood in the normal LV is age-dependent due to physiological changes in the degree of apical penetration of the filling waves.

## Introduction

Cardioembolic stroke is a major source of mortality and disability worldwide and blood stasis one of its major determinants (Adams et al., 1986). Left ventricular (LV) function has evolved to maximize mechanical efficiency and ensure organ perfusion at a low cost of energy and filling pressures. An additional requirement of blood flow homeostasis is avoiding the risk of thrombosis inside cardiac chambers.

Blood flow arrangement inside the LV ensures an effective washout and protects blood elements against high shear stresses (Kilner et al., 2000; Lowe, 2003). However, blood does not transit the LV following a first-in-first-out rule; even in normal hearts, a significant fraction of the blood entering the LV is not ejected during the ensuing systole (Bolger et al., 2007; Eriksson et al., 2011). This fraction increases in diseased hearts, potentially resulting in blood stagnation and eventually thrombosis inside the chamber (Eriksson et al., 2011; Hendabadi et al., 2013; Rossini et al., 2017). Thus, understanding the physiological foundations of intraventricular blood transit is of major clinical relevance. Furthermore, although avoiding stasis and shear stress is a major requirement for the design of LV assistance devices and valvular prostheses (Alemu and Bluestein, 2007; Steinlechner et al., 2009), the reference values of these indices have never been reported in the normal heart. Blood stirring is driven mainly by diastolic flow (Seo and Mittal, 2013), and age is a major determinant of filling (Schmitz L. et al., 1998; Strait and Lakatta, 2012). Hence, one may hypothesize that blood transit in the LV may be, to some extent, age-dependent. Although intuitive, to our knowledge, this hypothesis has not been tested and the normal indices of blood transit and shear stresses in the healthy heart have never been reported.

We have recently implemented an ultrasound-based imaging method to quantify blood stasis, readily applicable to the clinical setting, based on mapping the residence time of blood inside the LV (Bermejo et al., 2015; Rossini et al., 2016; Martinez-Legazpi et al., 2018). Residence time accounts for the time that a blood volume element remains inside the chamber. This methodology has shown an good predictive value to address mural thrombosis after an acute myocardial infarction (Martinez-Legazpi et al., 2018). Platelet activation is triggered by the intensity of shear stress, as well as by the duration of shear acting on the platelets (Ramstack et al., 1979). Therefore, adding the capacity of quantifying the exposure of blood to shear forces renders this imaging technology particularly well-suited for quantifying thrombosis-related metrics of flow homeostasis.

On this basis, we designed the present study to quantify the residence time and shear stresses of blood in the LV in a large sample of healthy subjects of different ages. We also aimed to address how blood transit is conditioned by age-related changes in diastolic function. We based the assessment of chamber washout on time-evolving maps of blood residence time in the LV. From these residence time maps we derived, two interlaced indices: (1) the global residence time (*RT*), and (2) an analysis of the evolution of the blood partial volumes with high *RT* values inside the chamber. We based the assessment of blood shear stresses on time-evolving maps of the shear index, *S*, an index that accounts for the blood transport of the largest values of the shear stresses. From these shear maps we derived another two interlaced indices: (1) the averaged *S* that takes place in the LV, and (2) the topological mapping of the regions with highest *S* within the chamber.

## Methods

### Study Population

We prospectively studied 149 healthy volunteers free of heart or vascular disease as determined by their clinical records and their echocardiographic examinations. Subjects were recruited in an open call in our institution to health professionals, their known and their relatives. Additionally, newborns and young children were recruited by inviting their parents or legal tutors to participate at the time of a programmed revision by their pediatrician. Height, weight and body surface area were recorded. Exclusion criteria were: (1) absence of sinus rhythm, (2) an abnormal ECG tracing, (3) any history of cardiovascular disease, obesity, or hypertension, (4) evidence of echocardiographic abnormalities, and (5) an unsuitable echocardiographic window. The study sample size was specified as a function of 8 different age groups: Group A: [0–1) years, Group B: [1–5) years, Group C: [5–10) years, Group D: [10–17) years, Group E: [17–30) years, Group F: [30–50) years, Group G: [50–65), and Group H ≥ 65 years. Group size was a priori established based on age-related changes previously described on cardiac size and diastolic function. The Institutional Ethics Committee for Clinical Research approved the study and all subjects (or their parents or legal tutors) signed written informed consent.

### Image Acquisition and Analysis

Comprehensive echocardiographic examinations were performed using Vivid-7 or Vivid-i scanners and phase-array (2- to 4-MHz or 6-MHz) transducers (General Electric Healthcare). Pulsed-wave Doppler spectrograms were obtained at the level of the mitral tips and the LV outflow tract. Ventricular sphericity was computed as the ratio between LV long and short axes measured from the apical four-chamber and parasternal long-axis views at end-diastole, respectively. The temporal events of the cardiac cycle —aortic valve opening (AVO), aortic valve closing (AVC), mitral valve opening (MVO), peak E wave velocity, A wave onset, peak A wave velocity, and mitral valve closure (MVC)— were measured from spectral Doppler recordings using EchoPac software (version 110.1.2, General Electric Healthcare) and forwarded to the fluid mechanics solver (echo-CDV, see below) (Garcia et al., 2010). We defined early- and late-filling times (EFT & LFT) as the time from MVO to A-wave onset and from A-wave onset to MVC, respectively. Filling-time (FT) was defined as the time from MVO to MVC. Atrial filling fraction (AFF) was defined as A-VTI/(E-VTI + A-VTI), being E-VTI and A-VTI the velocity time integrals of the early and late filling waves, respectively (Schmitz L. et al., 1998). Other conventional Doppler-echocardiographic data were recorded and measured following current recommendations (Lang et al., 2015).

### Residence Time and Shear Index Mapping

**Figure 1:**
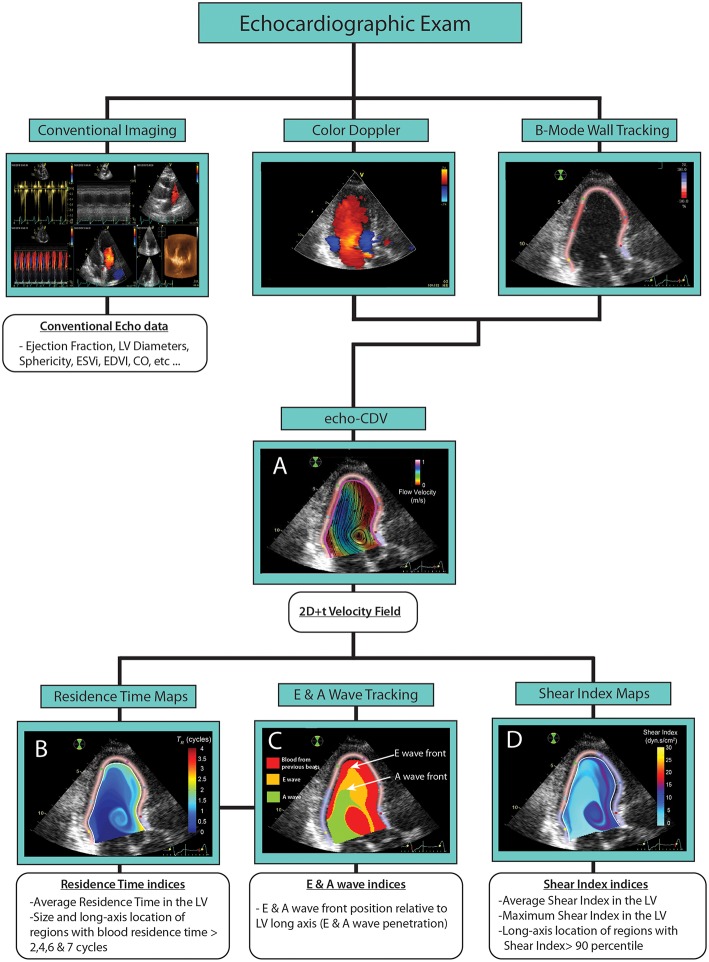
Methods of image acquisition and processing. **(A)** Echo CDV velocity maps. **(B)** Residence Time maps. **(C)** E & A waves tracking maps. **(D)** Shear index maps.

To quantify both LV blood wash out and hemodynamic stresses we used previously published methods. First, we reconstructed the 2D time-dependent (2D+t) blood velocity field in the LV using color-Doppler Velocimetry (echo-CDV) (Figure 1A). For this purpose, we integrated the flow continuity equation by using the radial velocities obtained from the color-Doppler data and the evolution of the LV myocardial wall as boundary conditions. The latter was obtained using speckle-tracking software (EchoPac, BT08, General Electric Healthcare). This method has been widely described, validated and applied in both the experimental and clinical settings, and has good spatial (~0.5 mm) and temporal (~5 ms) resolutions (Garcia et al., 2010; Hendabadi et al., 2013; Bermejo et al., 2014; Stugaard et al., 2015).

From the 2D+t velocity data we integrated two forced advection equations with the twofold purpose of mapping and quantifying (1) the residence time (*RT*) that infinitesimal blood elements spend inside the LV (Mangual et al., 2012; Hendabadi et al., 2013; Rossini et al., 2016), and (2) the accumulation of strong shear events along particle trajectories, quantified by a scalar, the Shear Index .*S* (Vu et al., 2019). The governing equations for *RT* and *S* are

(1) $$\begin{array}{r} \frac{\partial RT}{\partial t}+\nabla .\left(\vec{v}RT\right)=1, \end{array}$$

(2) $$\begin{array}{r} \frac{\partial S}{\partial t}+\nabla .\left(\vec{v}S\right)={\overset{∙}{\gamma }}^{\alpha } \end{array}$$

where $\vec{v}\;$ and $\overset{∙}{\gamma }\;$ are, respectively, the echo-CVD velocity field and the shear-rate of the fluid at each point of space and time inside the LV. Specifically, shear-rate was defined as $\overset{∙}{\gamma }={\left[trace\left(\Sigma^{2}\right)\right]}^{1/2}\;,\;$ where Σ is the symmetric part of the velocity gradient tensor, and multiplied by the Newtonian blood viscosity value (3.8 dyn·s/cm). Both *RT* and *S* were calculated in terms of absolute time and scaled to the cardiac cycle of each subject to normalize for age-related changes in heart rate. The forcing term in equation (2), ${\overset{∙}{\gamma }}^{\alpha },$ is a commonly used power-law model of shear-mediated platelet activation (Fraser et al., 2012). It balances the effects of shear stress intensity and cumulative shear exposure time by the value of the exponent α. Low values of α emphasize exposure time over shear intensity [in fact, when α = 0 equation (2) is identical to the *RT* equation (1)], whereas high values of α emphasize strong shear events over exposure time. The value of α = 2 was estimated by fitting the power-law model to the experimental data compiled by Hellums' as recently reported (Hellums, 1994; Vu et al., 2019). Conveniently, *S* keeps the same dimensions as the shear rate $\overset{∙}{\gamma }\;$ for this choice of the exponent.

The *RT* and *S* fields were integrated for 8 cardiac cycles from the first MVO to obtain time-averaged global indices of residence time and shear index within the chamber, $\bar{RT}$ and $\bar{S}$ (Figures 1B–D). Additionally, we determined the maximum (*S*_*max*_) values of and *S* in the LV at same time. The 15 studies showing longest $\bar{RT}$ were selected for reprocessing using runs of 16 cycles to ensure that results did not change using a longer temporal window

From the *RT* and *S* maps at the end of the calculation, we identified connected blood regions with *RT* > *2, 4, 6*, and *7* cycles, and with *S* > the 90th percentile of *S* in each subject. The size and the long-axis location (normalized between 0 —the mitral annular plane, and 1 —the apex) of these regions were stored and further analyzed. Regions smaller than 5% of LV end-diastolic volume (area in 2D) were dismissed. Based on forwarded temporal events, we thresholded *RT* maps during the diastole of the first beat to isolate the blood fractions carried by E- and A- waves (Rossini et al., 2017) (Figure 1C). The front location of these volumes relative to the normalized long-axis of the LV was measured to determine the degree of penetration in the LV of E and A-waves. The full processing method is summarized in Figure 1 and Supplementary Video 1.

To address the reliability of the method, we randomly selected *n* = 8 subjects to blindly acquire echocardiographic images by two independent investigators and reacquire by one of them. All processing was carried out thrice: calculation of 2D+t flow velocity fields, *S* and *RT* maps, event-time identification and final metrics calculation. The full test-retest reproducibility was good for *RT* and moderate for *S*: intra-class correlation coefficient > 0.75 for all *RT* indices, and between 0.40 and 0.80 for *S* metrics (Supplementary Table 1).

### Statistical Analysis

Variables are described as median, IQR range, and 5 and 95% quantiles. All the statistical analyses were performed pooling data from all the participants, disregarding the recruitment group information. Data were analyzed as a function of age unless otherwise specified. To fit the data, we applied ordinary least-squares univariate and multivariate linear and quadratic correlation analyses. We calculated correlation and determination (non-linear and multivariate fittings) coefficients where appropriate. Multivariate models were selected by backwards-stepwise regression based on Akaike's information criterion. To assess the nature of the relationships between (1) diastolic properties (2) age and (3) *RT* indices, we used mediation analysis with single/multiple mediators and bootstrap (*n* = 500 replicates) (VanderWeele and Vansteelandt, 2014). Mediation and direct effects and their 95% CIs were calculated for these analyses. Statistical significance was established at the *p* < 0.05 level. Statistical analyses were performed using *R* version 3.3.1.

## Results

We prospectively studied 149 healthy volunteers from 26 days to 80 years old. Demographic and echocardiographic data can be found in Table 1. Median age was 14 years old (IQR: 3–49, range: 26 days−80 years), and 47% of subjects were women.

**Table 1:** Demographic and echocardiographic data.

	**Group A**	**Group B**	**Group C**	**Group D**	**Group E**	**Group F**	**Group G**	**Group H**
Age interval (years old)	[0–1)	[1–5)	[5–10)	[10–17)	[17–30)	[30–50)	[50–65)	≥ 65
*N*	22	23	21	15	13	18	24	13
Sex (Female %)	9 (40%)	11 (47%)	12 (57%)	7 (46%)	7 (53%)	8 (44%)	8 (33%)	9 (69%)
Age (years)	0.26 (0.17–0.5)	2.57 (2.1–3.5)	6.76 (6.2–8.16)	14.36 (11.5–15)	21 (19–28)	37.5 (31–48)	55 (52–59.25)	75 (68–78)
Weight (Kg)	6.1 (5.0–7.5)	14.2 (12.3–16.2)	23.2 (20.7–26.7)	51 (34–55.5)	70 (60–75)	75 (61–85)	72 (67.7–77.2)	66 (59.5–70.2)
Height (m)	0.61 (0.60–0.70)	0.95 (0.90 – 1.0)	1.19 (1.15–1.27)	1.55 (1.46–1.6)	1.75 (1.72–1.79)	1.72 (1.66–1.79)	1.68 (1.60–1.70)	1.60 (1.50–1.66)
Heart Rate (b.p.m)	137 (127–142)	99 (93–108)	84 (75–92)	66 (61–72)	61 (58–69)	60 (56–66)	58 (53–61)	67 (64–71)
Body surface area (m^2^)	0.31 (0.27–0.36)	0.61 (0.54–0.66)	0.89 (0.82–0.93)	1.48 (1.20–1.61)	1.85 (1.75–1.93)	1.88 (1.70–2.03)	1.79 (1.71–1.86)	1.70 (1.61–1.78)
EDVi (mL/m^2^)	47 (40–55)	45 (38–53)	50 (45–58)	56 (51–67)	56 (53– 67)	50 (44–54)	44 (39–53)	40 (37–47)
ESVi (mL/m^2^)	15 (10–22)	15 (12–19)	19 (16–20)	19 (17–26)	21 (19–27)	19 (16–20)	17 (14–20)	16 (15–18)
Ejection fraction (%)	66 (61–71)	64 (62–67)	62 (60–66)	65 (62–66)	61 (60–64)	64 (60–65)	62 (60–67)	63 (59–66)
Stroke volume index (ml/m^2^)	32 (26–36)	29 (25–35)	30 (29–37)	37 (31–42)	36 (32–39)	31 (28–35)	29 (24–33)	26 (24–28)
Cardiac index (L/min/m^2^)	2.5 (2.1–2.9)	2.5 (2.1–3.3)	2.2 (1.9–2.5)	2.1 (1.8–2.3)	2.2 (1.9–2.8)	1.8 (1.6–2.3)	2.3 (2.1– 2.9)	2.7 (2.0 – 4.0)
LV EDD (cm)	2.3 (2.1–2.4)	3.0 (2.8–3.2)	3.5 (3.3–3.7)	4.1 (3.9–4.6)	4.7 (4.5–5.0)	4.7 (4.1 – 5.0)	4.5 (4.3–4.8)	4.3 (4.1–4.7)
LV length (cm)	4.2 (3.6–4.2)	5.6 (5.5–5.9)	6.5 (6.3–7.0)	7.9 (7.4–9.2)	9.1 (8.5–9.4)	8.8 (8.4 – 9.0)	8.2 (7.6–8.7)	7.5 (7.3–8.1)
LV sphericity	0.48 (0.47–0.61)	0.54 (0.51–0.56)	0.55 (0.5–0.56)	0.52 (0.49–0.53)	0.53 (0.50–0.54)	0.51 (0.49–0.56)	0.54 (0.52–0.58)	0.57 (0.53–0.62)
E–wave peak velocity (m/s)	0.97 (0.80–1.02)	1.01 (0.93–1.10)	1.01 (0.93–1.13)	0.9 (0.84–0.99)	0.83 (0.7–0.98)	0.78 (0.67–0.84)	0.7 (0.57–0.75)	0.53 (0.48–0.69)
A–wave peak velocity (m/s)	0.76 (0.66–0.94)	0.65 (0.49–0.75)	0.46 (0.41–0.49)	0.4 (0.36–0.42)	0.45 (0.42–0.55)	0.44 (0.37–0.47)	0.60 (0.56–0.67)	0.82 (0.69–0.92)
$\bar{RT}$ (cycles)	1.0 (0.7–1.2)	1.1 (0.9–1.5)	1.5 (1.1–1.7)	1.8 (1.4–2.2)	1.9 (1.7–2.2)	1.6 (1.3–2.2)	1.7 (1.3–2.2)	1.5 (0.9 −1.7)
$\bar{S}$ (dyn·s/cm^2^)	4.4 (3.6–5.5)	5.2 (4.1–6.4)	6.6 (5.1–7.9)	5.4 (4.9–6.8)	4.9 (4.0–5.6)	4.9 (3.8–6.7)	5.0 (4.2–6.7)	5.7 (4.4–8.1)
*S_*max*_*(dyn·s/cm^2^)	18.9 (15.0–24.3)	19.2 (14.1–24.3)	20.9 (15.8–25.5)	18.7 (15.3–24.5)	18.3 (11.7–20.8)	14.8 (11.9–20.2)	16.7 (13.1–21.2)	18.7 (13.6–28.3)

*EDVi, End Diastolic Volume Index; ESVi, End Systolic Volume index; CI, Cardiac Index; LV EDD, LV End Diastolic Diameter; $\bar{RT}$, Averaged Residence time in the LV; $\bar{S}$, Averaged value of cumulative shear of blood inside the left ventricle; S_max_, Maximum value of cumulative shear of blood inside the left ventricle. Values are expressed as median (IQR)*.

### Average Residence Time and Shear Index

**Figure 2:**
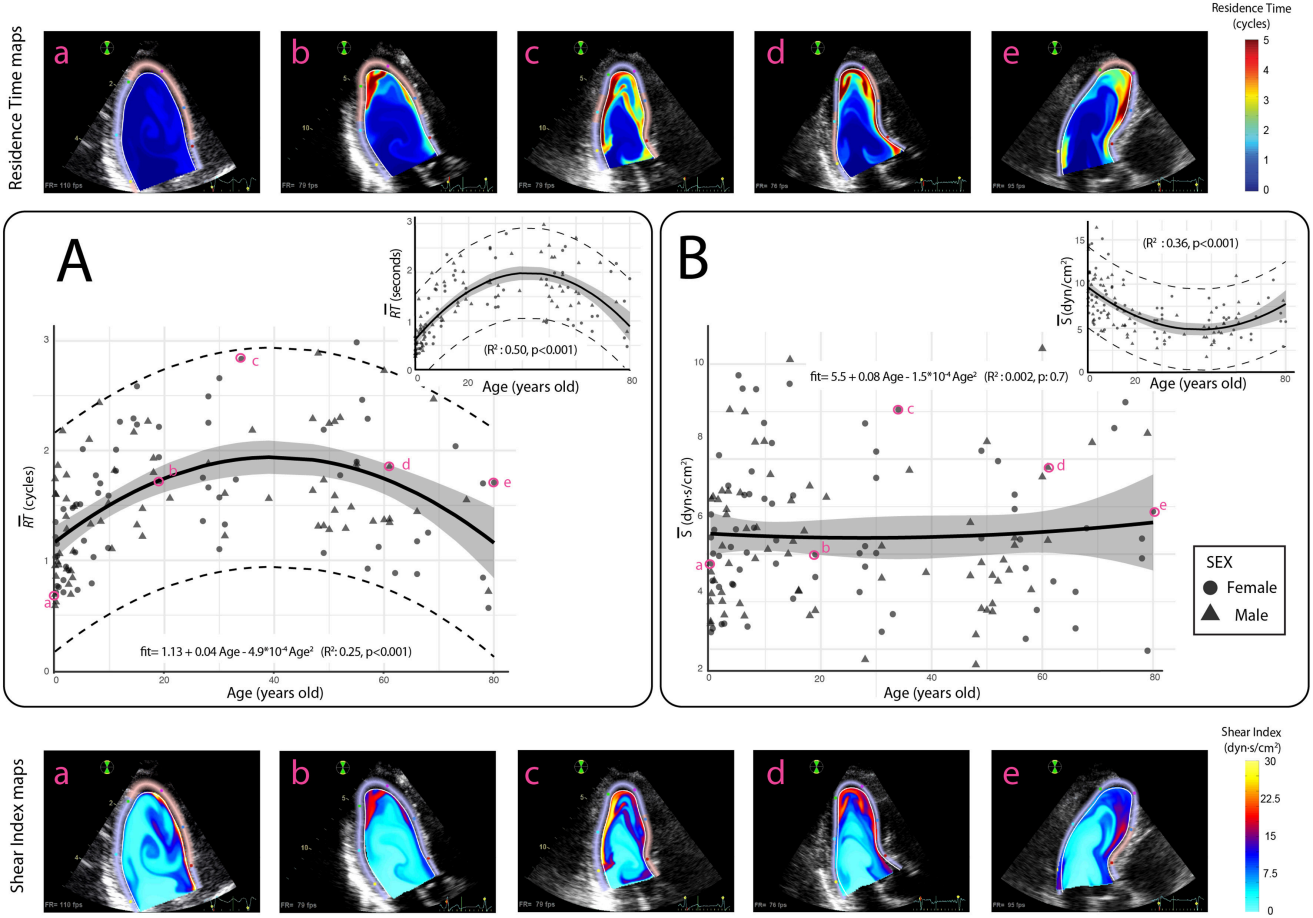
Relationships between age and averaged residence time ($\bar{RT}$) and shear index ($\bar{S}$) of blood inside the LV. **(A)** Age variation of average $\bar{RT}$ (scaled in cycles). Insert: $\bar{RT}$ expressed in seconds. **(B)** Age dependence of $\bar{S}$ (scaled in dyn·s/cm^2^). Insert: $\bar{S}$ expressed in dyn/cm^2^. Symbols account for sex. The **solid black lines** indicate the quadratic fits, the **shadowed areas** indicate the 95% confidence limits for the fittings and **dashed lines** indicate 95% prediction limits. Upper panels (a-e): RT maps and lower panels (a-e): S maps for selected patients (in magenta).

The median $\bar{RT}$ for the full study population was 1.5 cycles. Importantly, *RT* was lower than 3 cycles in all subjects, and lower than 1 cycle in 32 (21% of study population). The $\bar{RT}$ followed an inverse U-shaped relationship with age (Figure 2A and Table 1), sharply rising from 1.0 cycle in the newborn child (62–192 days old) to 1.8 cycles in adolescents (10–17 years old). The longest $\bar{RT}s$ were found between 17 and 50 years old (1.6 to 1.9 cycles), becoming shorter thereafter and reaching a median value of 1.5 cycles by the age of 65. This non-linear trend of the $\bar{RT}$ vs. age relationship was well captured by a quadratic fit ($\bar{RT}=$ 1.13 + 0.04·Age−4.9·10^−4^·Age^2^, R^2^ = 0.25, *p* < 0.001). There was a relatively wide inter-individual variation within each age group, with an IQR (5–95% quantiles) varying from 0.7 to 1.2 (0.6–1.7) cycles in the newborns to 1.3–2.1 (1.1–2.8) cycles in the 30-to-40-year-old group. There was no significant effect of sex on $\bar{RT}$, neither in a univariate fitting (*p* = 0.7) nor in the age-adjusted model (*p* = 0.5). The median $\bar{S}$ and *S*_*max*_ values for all studied subjects were 5.3 (4.1–6.6) dyn·s/cm^2^ and 18.5 (13.7–23.4) dyn·s/cm^2^, respectively, showing no changes with age when corrected for heart-rate (Figure 2B and Table 1).

### Region Analysis

**Figure 3:**
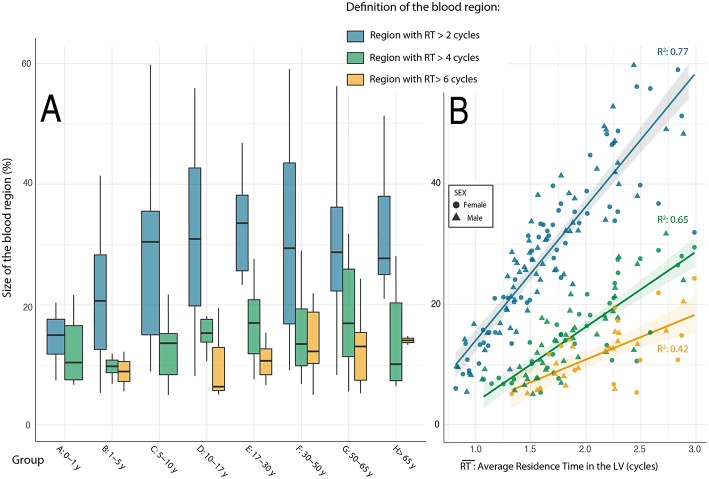
Relationship between age groups and the size of blood volumes with longest residence time. **(A)** Changes in the size of blood regions with age groups. **(B)** Relations between average residence time of blood in the LV and the size of retained blood volumes.

Regions of blood with *RT* > 2 cycles were found in 120 subjects (80%) with a median (IQR) size equal to 28.1 (19.2–36.3)% of global LV area. Likewise, blood regions with *RT* > 4 and 6 cycles were found in 77 (52%) and 33 subjects (22%); their median (IQR) sizes were 13.9 (9.5–18.8)% and 12.2 (8.0 −14.8)% of LV area, respectively (Figure 3A). Only 2 subjects (1.3%) showed regions of *RT* > 7 cycles, and these regions were small (< 8.0% of LV area). Regions of *RT* ≥ 8 cycles were not found in any subject (confirmed in the 16-cycle runs). Long-RT regions were located most apically in the chamber; the median (IQR) centroid was located at 0.65 (0.60–0.70), 0.73 (0.68–0.78), and 0.80 (0.71–0.84) along the normalized LV long-axis for the regions with *RT* of 2, 4, and 6 cycles, respectively. The size of long-RT regions correlated with $\bar{RT}$ (*R*^2^ = 0.77, *R*^2^ = 0. 65, and *R*^2^ = 0.42 for the regions with *RT*> 2, 4, & 6 cycles, respectively. Figure 3B). The regions of blood with *S* beyond its 90th percentile were also located apically with their normalized LV long-axis centroid position at 0.67 (0.56–0.76). Age variations of size and apical position of blood regions with longest *RTs* and highest *S* values are reported in Supplementary Table 2.

### The Determinants of Residence Time and Shear

**Figure 4:**
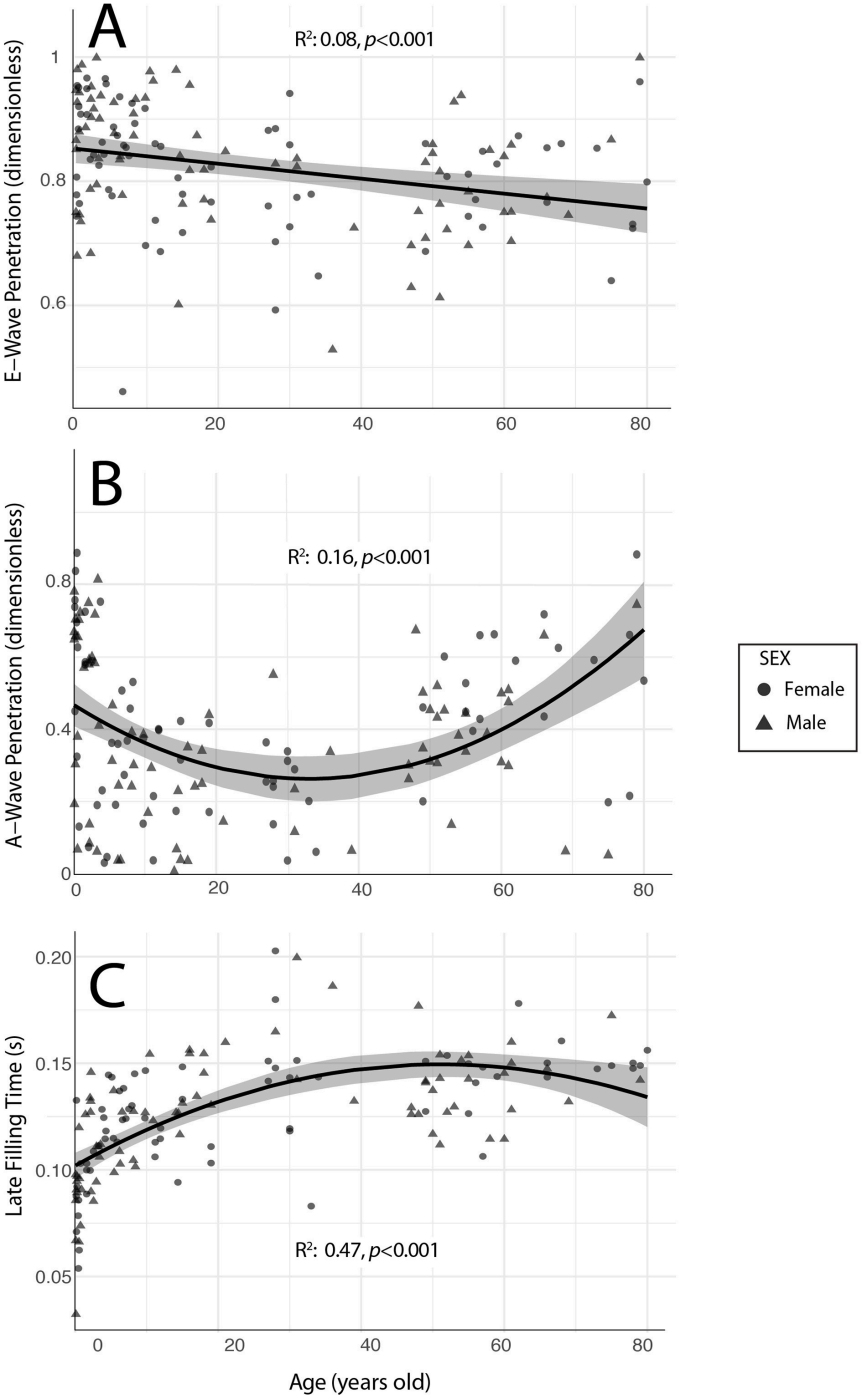
Relationship between age and the diastolic properties related to ($\bar{RT}$): E-wave penetration **(A)**, A-wave penetration **(B)** and Late Filling time **(C)**. Symbols account for sex.

**Figure 5:**
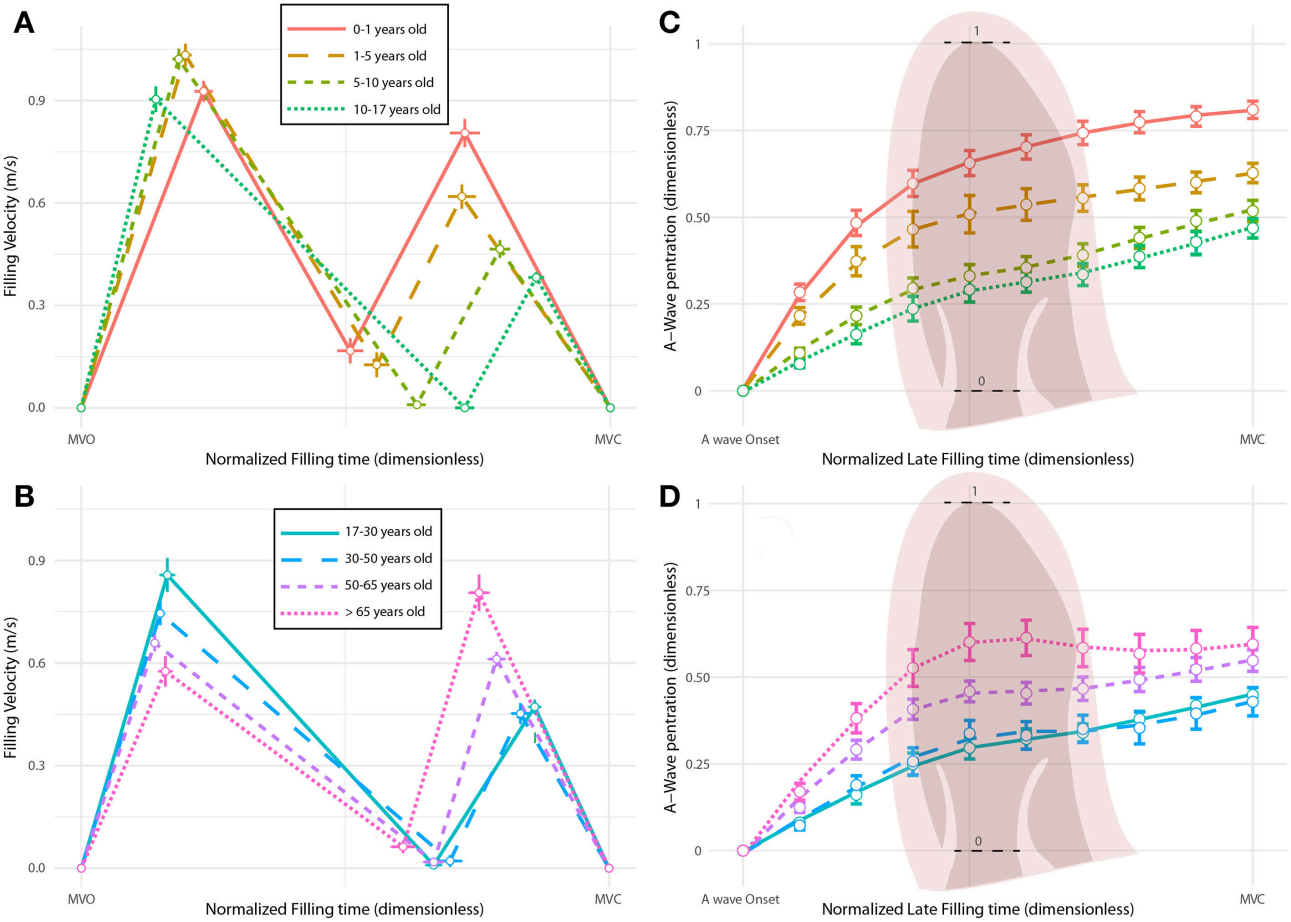
Age-related changes in filling profiles and late-filling wave penetration. (**A,B)** E and A wave patterns during filling. Horizontal axes represent normalized filling time from mitral valve opening (MVO) to mitral valve closing (MVC). **(C,D)** Tracking of the A-wave penetration along the LV long axis during the late filling phase, from A-wave onset to mitral valve closing (MVC). Horizontal axes represent the normalized late filling time and vertical axes the normalized long-axis length from the base to the apex. Colors account for age groups.

By univariate analyses, $\bar{RT}$ correlated with heart rate, LV long-axis dimension, penetration of E and A-waves, as well as with both early-filling and late-filling times (Table 2). Multivariate regression analyses showed that $\bar{RT}$ was inversely and independently related to E-Wave penetration (*p* < 0.001), A-Wave penetration (*p* = 0.002) and late-filling time (*p* < 0.001; Global $R_{\text{adj}}^{2}$ = 0.48, *p* < 0.001, Table 3). Heart-rate scaled $\bar{S}$ was found to be invariant to changes in age or diastolic properties. Mediation analysis showed that the combination of E-wave penetration, A-wave penetration and late-filling time accounted for 99.8% of the effect of age on $\bar{RT}$ (95% CI of the mediation effect: 99.0–100%, *p* < 0.001). This result can be explained by the relationship of the three former variables with age: E-wave penetration (*R*^2^ = 0.08, *p* < 0.001, Figure 4A), A-Wave penetration (*R*^2^ = 0.16, *p* < 0.001, Figure 4B) and late-filling time (*R*^2^ = 0.47, *p* < 0.001, Figure 4C). In turn, E-wave and A-wave penetration correlated with E-wave and A-wave velocities (*R*^2^ = 0.09, *p* < 0.001 and *R*^2^ = 0.30, *p* < 0.001, respectively) as well as with LV long-axis length (*R*^2^ = 0.11, *p* < 0.001 and *R*^2^ = 0.14, *p* < 0.001, respectively). Age-related differences in filling flow patterns and A-wave penetration during diastole are shown in Figure 5. Values of the remaining diastolic indices are shown in Supplementary Table 3.

**Table 2:** Univariate analysis of the relationship between $\bar{RT}\;$ and diastolic indices.

	**Model**	***R^**2**^***	***p***
Age	Quadratic	0.25	<0.001
Heart rate	Quadratic	0.16	0.002
LV ejection fraction	Linear		0.2
Stroke volume index	Linear		0.4
Cardiac index	Linear		0.6
LV end-diastolic volume Index	Linear		0.2
LV length	Linear	0.16	<0.001
**EARLY-FILLING PROPERTIES**			
E-wave-velocity	Linear	0.10	<0.001
E-wave-penetration	Linear	0.37	<0.001
E-wave time-velocity integral	Linear		0.2
Early filling time	Quadratic	0.16	<0.001
**LATE-FILLING PROPERTIES**			
A-wave-velocity	Quadratic		0.6
A-wave-penetration	Quadratic	0.17	<0.001
A-wave time-velocity integral	Linear		0.2
Late filling time	Quadratic	0.12	<0.001
Atrial filling fraction	Linear		0.1
E/A ratio	Linear		0.9

**Table 3:** Multivariate analysis between $\bar{RT}$ and diastolic indices.

	**Model**	**β**	**β (std error)**	***p***
E-wave-penetration	Linear	−3.02	0.36	<0.001
A-wave-penetration	Linear	1.31	0.56	0.02
	Quadratic	−2.08	0.69	0.002
Late-filling time	Linear	18.33	6.81	<0.001
	Quadratic	−65.74	27.43	0.01

*Global R^2^adj: 0.48; p < 0.001*.

## Discussion

To our knowledge, this is the first systematic description of residence time and shear exposure of blood in the normal human LV. By studying a large (*n* = 149) population of normal subjects, we demonstrate the age dependence of global and regional indices of residence time. We find that this dependence is explained by physiological age-related differences in filling flow. We also show that blood crosses the normal LV experiencing low shear stresses, being naturally protected against platelet activation. These findings have important implications for understanding ventricular flow dynamics in health, disease and device design.

### Intracardiac Blood Flow and Cardioembolism

Cardioembolic stroke is a major source of mortality and disability worldwide, and hemodynamic abnormalities are a recognized major risk factor for intracardiac thrombosis (Adams et al., 1986). A combination of blood stasis and increased shear stresses is known to be one of the situations most prone to thrombogenesis (Lowe, 2003). Importantly, if sustained during long periods, even moderate values of shear stress (i.e., in the range of 20 dyn/cm^2^) have been shown to activate platelets *in vitro* (Hellums, 1994; Shen et al., 2008). The averaged values we obtained in the normal heart are four-fold lower than the reported thresholds that trigger platelet activation. However, in given cardiac conditions, these metrics of flow mediated pro-thrombosis are expected to change considerably.

In atrial fibrillation, the major source of cardio embolism has been classically attributed to the combination of blood stasis and abnormal shear stresses that leads to thrombi formation in the vicinity of the atrial appendage (Stoddard et al., 1995). In acute myocardial infarction, an increased risk of embolism is found when endocardial damage is accompanied by an alteration of blood flow trajectories that impair blood washout (Delewi et al., 2012). In non-ischemic dilated cardiomyopathy intraventricular stasis is assumed to be the main risk factor related to mural thrombosis (Bakalli et al., 2013). Assistance devices and prosthetic valves always disrupt intraventricular flow to some degree, which may occasionally lead to blood stagnation and/or to a remarkable increase in blood shear stresses (May-Newman et al., 2013; Rossini et al., 2017). The lack of suitable quantitative methods has heavily limited addressing the alterations in flow homeostasis in these conditions.

Until now, intracavitary stasis has been qualitatively assessed by spontaneous contrast using B-mode echocardiography (Black et al., 1991). Due to the low reproducibility and subjectivity of this technique, anticoagulation therapies have been tailored in patients at risk based only on clinical and conventional imaging variables such as EF. Unfortunately, these approaches have proved to be of limited clinical benefit in ischemic and non-ischemic dilated cardiomyopathies (Koniaris and Goldhaber, 1998). A non-invasive quantification of blood stasis and shear stresses using quantitative metrics such as the ones presented in this work is particularly promising to individualize the risk of intraventricular thrombosis in disease and guide device design (Martinez-Legazpi et al., 2018).

### Intraventricular Flow Homeostasis

Some of the methods used to infer intraventricular blood transport have been based on the integration of particle traces from 3D+t PC-MRI velocity fields (Bolger et al., 2007; Eriksson et al., 2011; Fredriksson et al., 2011). This approach has proved to be useful to understand flow volumes traveling through the ventricles. However, technical limitations preclude this methodology to characterize blood volumes using interrogation window of more than ± 2 beats, resulting in an incomplete description of blood washout in longer time spans. An alternative approach is to use a Eulerian framework, as the method used in this work, which is able to overcome these limitations (Mangual et al., 2012; Rossini et al., 2016).

Our data shows that blood washing is well-guaranteed in the normal LV at all ages. Average *RT* is bounded below 3 cycles, and practically all the blood within the LV is renewed after 6 to 7 cycles. Physiologically, this is achieved because, despite not following a first-in-first-out rule, blood is replaced gradually in the LV as fresher blood flowing from the atrium stirs a fraction of the older blood and displaces it toward the outflow tract (Hendabadi et al., 2013). In the present study, we demonstrate that these processes are highly related to the apical propagation of filling waves, which in turn are modified by the physiological age-related changes in diastolic function. In earlier studies, volume interplays in the LV have been attributed mostly to early-diastolic flow phenomena. The vortex rings built during early filling entrains and stirs its surrounding blood before atrial contraction (Martinez-Legazpi et al., 2014; Bermejo et al., 2015). Furthermore, a reduced apical propagation of the E-wave has been suggested to impair blood wash out near the apex in patients with myocardial infarction (Harfi et al., 2017). By visualizing the interactions between the incoming and resident blood volumes, we demonstrate that the early and late filling phases have both similar relevance for clearing blood in the chamber.

In young children, as the heart grows, early filling (E-wave) velocities remain almost invariant, due to the progressive reduction of the intraventricular pressure gradients and the parallel increase of mitral valve area that take place during growth (Popovic et al., 2006). Consequently, at early ages the penetration of the E-wave filling wave along the long-axis of the ventricle remains relatively constant. Thus, if blood transport were to be conditioned solely by early-diastolic and diastatic flow phenomena the low values of blood residence time observed at early ages would be hard to explain. Although A-wave velocities are highest at early ages —due to the large atrio-ventricular pressure gradient mediated by atrial contraction— the time-velocity integral of the A-wave remains almost constant with age. Therefore, by injecting similar blood volume but in smaller cavities, late-filling blood volume propagates more apically in the LV at early ages, enhancing mixing and easing clearing. Of note, increased atrial contraction in subjects > 50 years old compensates the lower early-filling volumes caused by prolonged relaxation and increased chamber stiffness. Thus, the so-called impaired relaxation pattern of physiological aging may have a compensatory effect on intraventricular blood clearing.

Whether there are any teleological advantages of age-related changes in intraventricular transport is unknown. Theoretical requirements for optimal blood transport in the heart should include (1) maximizing mechanical efficiency (highest pump function at the lowest energetic cost), (2) avoiding blood stasis, (3) allowing the highest hemodynamic reserve during stress, and (4) providing the mechanical stimuli for an optimal heart development and biological hemostasis. These four factors are intrinsically intertwined and the ideal “solution” must optimize them together. For example, notice that the highest mechanical efficiency at rest would be obtained if all kinetic energy developed by filling is immediately transferred to ejection during systole (100% direct flow, meaning that the entire blood pool would be replaced each beat) (Bolger et al., 2007). However, this would require an LV ejection fraction of 1, discordant with a evolutionarily-preserved, basic trait of vertebrate cardiac function. The very fact that the LV ejection fraction is lower than unity entails the need for blood washing mechanisms. Thus, the existence of residual volumes that are barely set in motion by the incoming blood might positively impact the mechanical efficiency of the chamber, which could explain why residence time is observed to increase in patients with dilated cardiomyopathy (Rossini et al., 2016).

### Effects of Heart Rate

Shorter residence times at younger ages could be attributed to their faster heart rate. Therefore, one could hypothesize that increasing heart-rate in adults would be an effective way to facilitate blood clearing. However, tachycardia increases the velocity of the A-wave at the expense of fusing it with the E-wave (Oniki et al., 1992; Santhanakrishnan et al., 2016; Rossini et al., 2017). Thus, tachycardia and age-related changes in heart-rate induce different effects on blood mixing. In fact, shortening the AV delay does not generally improve intraventricular blood transport in patients undergoing cardiac resynchronization therapy (Rossini et al., 2017). Further investigations are needed, ideally combining experiments of pacing and exercise, to uncouple the effects on blood transport of heart rate, atrioventricular delay, inotropism, and lusotropism.

### Clinical Implications

Identifying a determinant role of filling-wave penetration on blood transport inside the LV has important consequences in the clinical setting. E-wave propagation is closely related to relaxation (Garcia et al., 2000) and therefore, diastolic dysfunction could lead to blood stasis in the chamber. We believe that this hypothesis could explain why a restrictive filling pattern is the most powerful factor related to silent brain infarcts in patients with non-ischemic dilated cardiomyopathy (Kozdag et al., 2006). In patients with atrial fibrillation, the absence of a late-filling phase impacts blood transport and could induce some degree of intraventricular stasis inside the LV. This may be an unreported mechanism of increased embolic risk beyond atrial appendage thrombosis, which has been shown to incompletely determine cardiac embolism in this condition (Brambatti et al., 2014). In terms of cardioembolic risk, the finding of highest *RT* in middle-age normal adults is intriguing. In addition, as the dispersion of normal *RT* was relatively high, some subjects show values not far from those of patients with myocardial infarction and mural thrombosis (Martinez-Legazpi et al., 2018). Maybe the *RT* of a given subject determines his/her idiosyncratic baseline cardioembolic risk in case he/she suffers a cardiac disease, or even cases of extremely long values of “normal” *RT* could be responsible for cardioembolic stroke of “undetermined origin.” We believe these issues should be addressed by future clinical trials.

The methodology used in the present study is readily applicable in the clinical setting to characterize blood stasis and shear stresses at other heart locations. Importantly, the method is well-suited to measure *RT* and *S* not only using ultrasound. Particularly promising is the implementation of 3-D residence time and shear topology maps in the four heart chambers based on phase-contrast MR velocity data (Bermejo et al., 2015; Rossini et al., 2016). Our results describe the physiological values of blood stasis and shear exposure in the LV, providing a reference framework for assessing intraventricular blood stasis and thromboembolic risk in disease. Moreover, our findings also emphasize the need for age-matched controls when comparing alterations of intraventricular flow dynamics.

### Limitations

Although the study population spanned a wide age range, the number of subjects in given age groups may seem small. Anticipating this limitation, we established sample-sizes of different age groups based on previously reported changes in cardiac size and diastolic function.

Intraventricular flow is highly 3-dimensional; however, we have analyzed it using a 2D method. Although this approach has been partially validated (Garcia et al., 2010; Bermejo et al., 2014), it has inherent benefits, such as a lower computational time and the widespread availability of echocardiography, its potential limitations must be weighted with the hypothesis of planar flow in the apical-long axis view of the LV. There is a lack of consensus whether total cumulated shear or time exposure to elevated shear beyond some threshold provides a more relevant index of platelet activation (Hellums, 1994). The present methodology could be easily adapted to map and quantify these additional metrics by modifying the forcing term in the right-hand side of equation (2). Echo-CDV imposes free slip conditions at the LV endocardium to obtain the azimuthal velocity component. Therefore, the shear values may be underestimated. Also, shear indices show moderate reproducibility. This is due to the intrinsically definition of these metrics, which involve the evaluation of the peak of the spatial-derivatives of the flow velocity, always adding some degree of noise. The shear index was calculated assuming blood to be a Newtonian fluid with fixed viscosity. However, blood viscosity is dependent of hematocrit, which was not measured in our study. Hematocrit depends on age and sex, and can also vary among individuals based on factors such as blood pressure and physical conditioning. Therefore, the values reported here must be interpreted with caution. In addition, notice that the method could be adapted to account for this source of individual variability.

Further studies using phase-contrast magnetic resonance data and/or computational fluid dynamics may help to clarify these issues. Ultimately, a common limitation of all methods that infer biomechanical metrics based on computation is that real validation is exceedingly difficult. However, these methods are still valuable since they allow researchers to compare the values of these metrics in different physiological and pathological conditions. Hence, establishing the age dependence of these metrics in normal subjects is important.

## Conclusions

In conclusion, energy conservation, blood residence time and shear stresses are intrinsically intertwined, so that optimal performance requires a balanced interplay between all physiological functions related to intracardiac flow dynamics. The average residence time of blood in the human LV is <3 cycles, and the blood pool of the LV is cleared in <6–7 cycles. The apical region of the LV is the most prone to stagnation, even in the normal heart. Blood is cleared at different rates depending on age, increasing from 1.0 cycle during the first months after birth, peaking at 1.8 cycles during the mid-age and then lowering again. The physiological changes in wave penetration explain the age-dependence of blood clearing. Nevertheless, there is a large individual variability neither explained by age nor sex. The degree of apical penetration of the early- and, particularly, the late-filling wave is the major determinant of blood mixing and clearing. Importantly, shear stresses do not change with age and physiological homeostasis protects against platelet activation.

## Data Availability

The raw data supporting the conclusions of this manuscript will be made available by the authors, without undue reservation, to any qualified researcher upon request.

## Ethics Statement

This study was carried out in accordance with the recommendations of the local Institutional Ethics Review Board. The protocol was approved by the local Institutional Ethics Review Board. All subjects or their parents gave written informed consent in accordance with the Declaration of Helsinki.

## Author Contributions

YB, PM-L, LR, CPV, RY, YM, DR-P, JdÁ, and JB participated in the conception and design as well as in the analysis, acquisition or interpretation of data. YB, PM-L, CPV, RY, MD, CM, JA, JdÁ, and JB edited, drafted, or revised the manuscript for important intellectual content; FF-A, JdÁ, and JB provided final approval.

### Conflict of Interest Statement

PM-L, LR, RY, JdÁ, and JB are inventors of a method for quantifying intracardiac blood stasis from imaging data under PCT patent application (WO2017091746A1). The remaining authors declare that the research was conducted in the absence of any commercial or financial relationships that could be construed as a potential conflict of interest.

## Abbreviations

MVO: Mitral Valve Opening
MVC: Mitral Valve Closing
AVO: Aortic Valve Opening
FT: Filling Time (from MVO to MVC) (s)
EFT: Early Filling Time (from MVO to A wave onset) (s)
LFT: Late Filling Time (from A wave Onset to MVC) (s)
AFF: Atrial Filling fraction (dimensionless)
*RT*: Blood residence time (cycles)
$\bar{RT}$: Average value of blood residence time in the left ventricle (cycles)
*S*: Shear index (dyn·s/cm^2^)
$\bar{S}$: Average value of Shear index of blood inside the left ventricle (dyn·s/cm^2^)
S_*max*_: Maximum value of Shear index of blood inside the left ventricle (dyn·s/cm^2^).
