# Supplementary material for: Age-Dependence of Flow Homeostasis in the Left Ventricle
Source: Front Physiol. 2019 Apr 26;10:485. doi: 10.3389/fphys.2019.00485 (PMC6498893; doi:10.3389/fphys.2019.00485)
Supplement: Supplementary file 2 [file Data_Sheet_1.docx]

**Age dependence of left ventricular flow homeostasis**

**Yolanda Benito, DCS, DVM^1^, Pablo Martinez-Legazpi, MEng, PhD^1^, Lorenzo Rossini, AEng, PhD^2^ , Candelas Pérez del Villar, MD, PhD^1^, Raquel Yotti, MD, PhD^1^, Yolanda Martín Peinador, MD , Daniel Rodríguez-Pérez, MPhys, PhD^4^, M. Mar Desco, MD, PhD^4^, Constancio Medrano, MD, PhD^1^, Jose Carlos Antoranz, MPhys, PhD^4^, Francisco Fernández-Avilés, MD, PhD^1^, Juan C. del Álamo, AEng, PhD^2,5^ and Javier Bermejo, MD, PhD^1^**

^1^Department of Cardiology, Hospital General Universitario Gregorio Marañón, Facultad de Medicina, Universidad Complutense de Madrid, Instituto de Investigación Sanitaria Gregorio Marañón, and CIBERCV, Madrid, Spain,

^2^Department of Mechanical and Aerospace Engineering. University of California San Diego, USA,

^3^Centro de Salud Goya, Dirección Asistencial Centro, Atención Primaria de Madrid, Spain

^4^Department of Mathematical Physics and Fluids, Facultad de Ciencias, Universidad Nacional de Educación a Distancia, Spain

^5^Institute for Engineering in Medicine. University of California San Diego, La Jolla, CA.

SUPPLEMENTARY MATERIAL

# **Supplementary Tables**

**SUPPLEMENTARY TABLE 1**: Intraclass correlation coefficient (Ric) and relative error (mean ± std) of the reproducibility study.

|  | **Interobserver** | | | **Intraobserver** | | |
| --- | --- | --- | --- | --- | --- | --- |
|  | R_ic_ | Relative Error (%) | | R_ic_ | Relative Error (%) | |
| $\bar{RT}$ | 0. 90 | 9 ± 25 | 0.77 | | 18 ±16 | |
| *Size _RT> 2_* | 0.90 | 31 ± 43 | 0.75 | | 16 ± 34 | |
| $\bar{S}$ | 0.79 | 6±34 | 0.42 | | 21±19 |  |
| *S_max_* | 0.62 | 12±29 | 0.66 | | 1±22 |  |

$\bar{RT}$*:* Averaged residence time in the LV, Size *_RT> 2_:* Size of blood regions with residence time > 2 cycles, $\bar{S}$*:* Averaged shear index in the LV. *S_max_*: Maximum shear index in the LV.

**SUPPLEMENTARY TABLE 2:** Normalized apical location of blood volumes with large residence time and shear stress.

|  | Group A  [0-1) years old | Group B  [1-5) years old | Group C  [5-10) years old | Group D  [10-17) years old | Group E  [17-30) years old | Group F  [30-50) years old | Group G  [50-65) years old | Group H  > 65 years old |
| --- | --- | --- | --- | --- | --- | --- | --- | --- |
|  |  |  |  |  |  |  |  |  |
| *Size _RT> 2_* (%) | 15 (12-18) | 21 (13-28) | 30 (15-36) | 31 (20-43) | 34 (26-38) | 29 (17-44) | 29 (22-36) | 28 (25-38) |
| *Size _RT> 4_* (%) | 10 (8-17) | 10 (9-11) | 14 (8-15) | 15 (14-18) | 17 (12-21) | 14 (10-19) | 17 (11-26) | 10 (7-20) |
| *Size _RT> 6_* (%) | --- | 9 (7-11) | --- | 6 (6-13) | 11 (8-13) | 12 (10-19) | 13 (8-15) | 14 (14-14) |
|  |  |  |  |  |  |  |  |  |
| X_C2_ | 0.65 (0.57-0.7) | 0.71 (0.63-0.84) | 0.62 (0.53-0.66) | 0.64 (0.59-0.68) | 0.65 (0.62-0.67) | 0.63 (0.6-0.67) | 0.67 (0.64-0.7) | 0.68 (0.65-0.69) |
| X_C4_ | 0.67 (0.61-0.72) | 0.82 (0.77-0.84) | 0.76 (0.67-0.79) | 0.73 (0.7-0.77) | 0.72 (0.69-0.77) | 0.73 (0.69-0.78) | 0.75 (0.7-0.78) | 0.69 (0.65-0.74) |
| X_C6_ | --- | 0.85 (0.84-0.85) | --- | 0.8 (0.72-0.84) | 0.84 (0.69-0.86) | 0.81 (0.79-0.82) | 0.79 (0.7-0.81) | 0.7 (0.69-0.72) |
| X_CS_ | 0.61 (0.42 - 0.69) | 0.62 (0.52 - 0.72) | 0.67 (0.49 - 0.73) | 0.69 (0.62 - 0.72) | 0.74 (0.64 - 0.78) | 0.67 (0.42 - 0.75) | 0.71 (0.59 - 0.79) | 0.59 (0.51 - 0.67) |

*Size _RT> 2_*: Size of blood regions with residence time >2 cycles, *Size _RT>4_*: Size of blood regions with residence time >4 cycles, *Size _RT> 6_*: Size of blood regions with residence time >6 cycles, X_C2_: Normalized apical location of blood regions with residence time >2 cycles, X_C4_: Normalized apical location of blood regions with residence time >4 cycles, X_C6_: Normalized apical location of blood regions with residence time >6 cycles, X_CS_: Normalized apical location of blood regions with Shear index over the 90^th^ percentile. Values are expressed in Median (IQR).

**SUPPLEMENTARY TABLE 3:** Other echocardiographic indices.

|  | Group A  [0-1) years old | Group B  [1-5) years old | Group C  [5-10) years old | Group D  [10-17) years old | Group E  [17-30) years old | Group F  [30-50) years old | Group G  [50-65) years old | Group H  > 65 years old |
| --- | --- | --- | --- | --- | --- | --- | --- | --- |
|  |  |  |  |  |  |  |  |  |
| E-Wave TVI (cm) | 7.1 (5.9 - 7.9) | 10.9 (9.3- 13.9) | 12.2 (10.1 - 14.0) | 13.7 (10.8 - 15.5) | 12.6 (11.3 - 14.8) | 11.2 (8.9 - 11.5) | 8.7 (8.1 - 9.6) | 7.6 (7.0 - 10.2) |
| E-Wave penetration (n.d.) | 0.87 (0.75 – 0.94) | 0.90 (0.84 – 0.94) | 0.87 (0.84 - 0.92) | 0.82 (0.75 -0.91) | 0.82 (0.76 - 0.85) | 0.74 (0.69 - 0.83) | 0.81 (0.75 - 0.85) | 0.80 (0.75 - 0.86) |
| Early filling time (s) | 0.11 (0.10 – 0.13) | 0.17 (0.14 – 0.22) | 0.27 (0.21 – 0.31) | 0.43 (0.37 – 0.45) | 0.38 (0.29– 0.44) | 0.51 (0.37 – 0.55) | 0.40 (0.37 – 0.47) | 0.30 (0.26 – 0.39) |
|  |  |  |  |  |  |  |  |  |
| A-Wave TVI (cm) | 4.8 (3.6 - 5.6) | 5.97 (4.9 - 6.8) | 3.9 (2.8 - 4.7) | 3.4 (3.2 - 3.7) | 3.9 (3.6 - 4.4) | 3.6 (3.2 - 4.6) | 5.7 (4.8 - 6.0) | 7.1 (6.4 – 8.0) |
| A-Wave penetration (n.d.) | 0.66 (0.38 - 0.72) | 0.58 (0.15 - 0.60) | 0.36 (0.24 - 0.39) | 0.21 (0.15 - 0.33) | 0.26 (0.24 - 0.36) | 0.29 (0.20 - 0.34) | 0.45 (0.37 - 0.51) | 0.59 (0.22 - 0.66) |
| Late filling time (s) | 0.09 (0.07 - 0.10) | 0.11 (0.10 – 0.13) | 0.13 (0.11 - 0.14) | 0.13 (0.12 – 0.14) | 0.15 (0.13 - 0.16) | 0.14 (0.13 - 0.15) | 0.14 (0.13 - 0.15) | 0.15 (0.15 - 0.15) |
| Atrial Filling Fraction (n.d.) | 0.39 (0.36 - 0.42) | 0.34 (0.28 - 0.37) | 0.24 (0.22 - 0.29) | 0.20 (0.19 - 0.24) | 0.23 (0.21 - 0.27) | 0.27 (0.22 - 0.31) | 0.39 (0.33 - 0.41) | 0.44 (0.42 - 0.47) |
|  |  |  |  |  |  |  |  |  |
| E/A Ratio | 1.2 (1.05 - 1.48) | 1.73 (1.42 - 2.04) | 2.26 (1.93 - 2.68) | 2.34 (2.1 - 2.55) | 1.74 (1.47 - 2.17) | 1.74 (1.38 - 2.07) | 1.02 (0.94 - 1.32) | 0.73 (0.62 - 0.80) |

Values are expressed in Median (IQR). n.d.: dimensionless
